# Influence of Target-Substrate Distance on the Transport Process of Sputtered Atoms: MC-MD Multiscale Coupling Simulation

**DOI:** 10.3390/ma15248904

**Published:** 2022-12-13

**Authors:** Guo Zhu, Qixin Du, Baijun Xiao, Ganxin Chen, Zhiyin Gan

**Affiliations:** 1 School of Mechanical & Electrical Engineering, Hunan City University, Yiyang 413000, China; 2 School of Mechanical Science & Engineering, Huazhong University of Science & Technology, Wuhan 430074, China

**Keywords:** molecular dynamics method, Monte Carlo method, magnetron sputtering, sputtered particles transport, incident energy distribution, incident angular distribution

## Abstract

A Monte Carlo (MC) and molecular dynamics (MD) coupling simulation scheme for sputtered particle transport was first proposed in this work. In this scheme, the MC method was utilized to model the free-flight process of sputtered atoms, while the MD model was adopted to simulate the collision between the sputtered atom and background gas atom so as to self-consistently calculate the post-collision velocity of the sputtered atom. The reliability of the MD collision model has been verified by comparing the computation results of the MD model and of an analytical model. This MC-MD coupling simulation scheme was used to investigate the influence of target-substrate distance on the transport characteristic parameters of sputtered Cu atoms during magnetron sputtering discharge. As the target-substrate distance increased from 30 to 150 mm, the peak energy of the incident energy distribution of deposited Cu atoms decreased from 2 to 1 eV due to the gradual thermalization of sputtered atoms. The distribution of differential deposition rate in unit solid angle firstly became more forward-peaked and then reversely approached the cosine distribution, which was agreed with the existing experimental observations. This work is expected to provide a more realistic simulation scheme for sputtered particle transport, which can be further combined with the MD simulation of sputtered film growth to explore the influence mechanism of process parameters on the properties of sputtered film.

## 1. Introduction

Due to its higher productivity and lower manufacturing cost, magnetron sputtering deposition has been extensively applied to the fabrication of metal, semiconductor, optical, and composite films [1,2,3,4]. During magnetron sputtering deposition, film particles undergo a series of collisions with background gas atoms before they are ultimately deposited onto the substrate, i.e., particle transport process, which affects the incident energy and angular distributions [5,6] of sputtered particles as they strike the substrate, and thus influences the performance of the deposited film. In the latest decades, the investigation of the particle transport process under various process conditions has attracted considerable attention [7,8,9,10,11,12,13,14,15,16] due to its importance in exploring the influence mechanism of process parameters on the properties of sputtered film.

The most challenging issue of particle transport research is the calculations of the post-collision velocities of sputtered particles. Nakano et al. [8] proposed a scheme to calculate the post-collision velocity of sputtered particles, but its complex coordinate transformation procedure limited its application. Based on classical binary collision theory, the post-collision velocity of sputtered particles can be expressed as a function of the relative velocity after collision. In order to simplify the calculation procedure, Yamamura et al. [9] further divided the relative velocity after collision into two orthogonal components (V_ep_ and V_eA_), but |V_eA_|/|V_ep_| was not equal to tan*θ*_com_ (*θ*_com_ is center-of-mass scattering angle). In most follow-up studies [10,11,12,13,14,15,16], the scattering angle of the sputtered particle after a collision is calculated by an approximate formula that is derived by supposing that the background gas atom is stationary before collision [17] and thus cannot precisely predict the post-collision velocity of the sputtered particle.

On the other hand, the molecular dynamics method as an atomic scale simulation tool has been employed to study the low-energy near-surface collision cascades in sputtering target material [18,19] and the high-energy collision cascades in diamonds under irradiation [20]. These simulations have accurately reproduced the collisions between energetic particles. In this work, an MC-MD coupling scheme for sputtered particle transport was first proposed, where the MD method was adopted to calculate the post-collision velocity of the sputtered atom. The MC-MD coupling method was employed to study the free-flight process of sputtered Cu atoms in the argon gas during DC magnetron sputtering discharge. In particular, the incident energy and incident angle distributions of the sputtered Cu atoms deposited onto the substrates under different target-substrate distances were investigated, and relevant mechanisms were discussed in detail. This work is expected to explore a more realistic and scale-matching simulation method for sputtered particle transport.

## 2. MC-MD Simulation Method

The present MC-MD model of sputtered particle transport is based on the following assumptions: (1) Sputtered particles are neutral atoms in the ground state [21]; (2) the sputtered particles only experience elastic collisions with background gas atoms due to their small concentration compared to that of background gas [22]; (3) The background gas is homogeneous and in thermal equilibrium at temperature *Tg* and pressure *P.* Based on these assumptions, the movement trajectory of a sputtered atom in the gas phase consists of a sequence of straight lines, which are ended by the elastic collisions with background gas atoms.

In this work, the MC-MD coupling method was employed to investigate the transport of sputtered Cu atoms in argon gas during a DC magnetron sputtering charge, which occurred in the space between the target and substrate in a parallel and coaxial configuration. The sputtering gas temperature, gas pressure, and sputtering target voltage were set to 300 K, 0.5 Pa, and −400 V, respectively. The simulation procedures are introduced as follows:

### 2.1. Initial Status Data of Sputtered Atom

**Figure 1:**
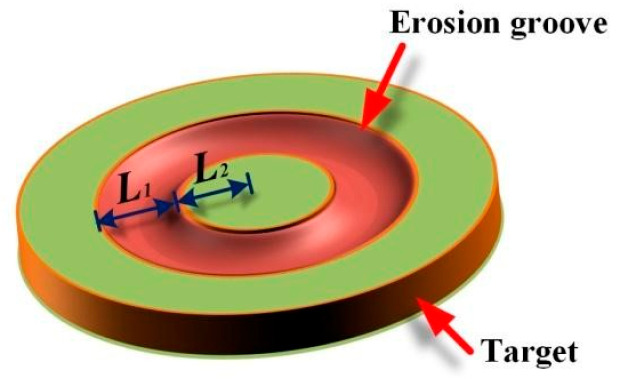
Sketch of the erosion groove on the circular planar target (*L*_1_ = 15 mm, and *L*_2_ = 5 mm).

The sputtered atoms are ejected from a 2-inch circular plane target. It is known that the sputtering of target material results in the formation of an annular erosion groove on the target surface. Figure 1 shows the geometric dimension of the erosion groove, whose profile is typically described by Gauss distribution [13]:

(1) $$f(d)=\exp[-{\left(d-\mu \right)}^{2}/2\delta^{2}]/\sigma \sqrt{2\pi }$$

As shown in Figure 1, the central circle radius and width of the erosion groove (*L*_1_) are 12.5 mm and 15 mm, from which the mean (*μ*) of 12.5 and standard deviation (δ) of 2.5 in Gauss distribution can be obtained. Accordingly, the sputtering probability of an atom located at a distance of *d* from the target center can be evaluated. Then, the emission position coordinates of a sputtered atom were generated by a random number *R*_1_, which is distributed uniformly between 0 and 1.

*x* = *d*cos(2π*R*_1_) (2)

*y* =*d*sin(2π*R*_1_) (3)

The initial energy distribution of sputtered atoms as they leave the target surface was assumed to obey the Thompson distribution [23], which is given as:

(4) $$f(E_{0})\propto \frac{1-\sqrt{\left(E_{\mathrm{coh}}+E_{0}\right)/\gamma E_{\mathrm{Ar}}}}{E_{0}^{2}{\left(1+E_{\mathrm{coh}}/E_{0}\right)}^{3}}$$

where *E*_b_ is the binding energy of target material (Cu); *E*_Ar_ is the bombarding energy of argon ion; *γ =* 4 *m*_g_*m*_s_*/*(*m*_g_ *+ m*_s_)^2^, *m*_g_*,* and *m*_s_ stand for the masses of background gas atom and sputtered atom, respectively. Then, the initial energy *E*_0_ of each sputtered atom was generated by the rejection algorithm [10,24].

The polar emission angles of sputtered atoms were chosen based on the angular distribution proposed by Yamamura et al. [25]. The number of atoms sputtered into the solid angle d*Ω* can be expressed as:

(5) $$dN=N_{t}f\left(\theta \right)d\Omega =N_{t}\cos\theta \left(1+\xi \cos^{2}\theta \right)d\Omega$$

where *N*_t_ is the total number of sputtered atoms, *θ* is the polar emission angle of the sputtered atom with respect to the target surface normal, and *ξ* is a fitting parameter according to the bombarding energy [25]. Due to symmetry, the azimuth emission angles of sputtered atoms were generated uniformly in [0, 2π].

### 2.2. MC Simulation of the Free-Flight Processes of Sputtered Atoms in Gas Phase

The collision between a sputtered atom and a background gas atom is typically assumed to be a random process complying with Poisson distribution [12,26]. The actual free-flight distance of the sputtered atom between two successive collisions can be calculated from:

*λ* = *λ*_m_|In*R*_2_| (6)

where *λ*_m_ is the mean free path; *R*_2_ is a random number uniformly distributed between 0 and 1.

In the following, the words ‘speed’ and ‘velocity’ are used for scalar and vector quantities, respectively. Since the energy of sputtered atoms can vary by several orders of magnitude, the energy-dependent *λ*_m_ is introduced and can be expressed as below [14]:

(7) $$\lambda_{m}=1/n\;\sigma =1/\left[n_{g}\pi P_{\max}^{2}\left(E_{\mathrm{com}}\right)\right]\left(v_{s}>5v_{p}\right)$$

(8) $$\lambda_{m}=1/n\;\sigma =1/\left[n_{g}\sigma \langle v_{r}\rangle \right]\left(\sqrt{3K_{B}T_{B}/m_{s}}<v_{s}<5v_{p}\right)$$

(9) $$\lambda_{m}=1/\left[n_{g}\pi {\left(r_{s}+r_{g}\right)}^{2}\sqrt{1+m_{s}/m_{g}}\right]\left(0<v_{s}<\sqrt{3K_{B}T_{B}/m_{s}}\right)$$

where *n*_g_ is the concentration of background gas; *σ* is the collision cross-section; *P*max is the maximum impact parameter corresponding to the minimum of center-of-mass scattering angle; *E*_com_ is the center-of-mass kinetic energy; *v*_p_ is the most probable speed of background gas atoms; *v*_r_ is the relative speed between the sputtered atom and background gas atom; *K*_B_ is Boltzmann constant; *T*_g_ is the temperature of background gas; *m*_s_ and *m*_g_ are the masses of sputtered and background gas atoms, respectively; *r*_s_ and *r*_g_ are the atomic radii of sputtered and background gas atoms, respectively.

**Figure 2:**
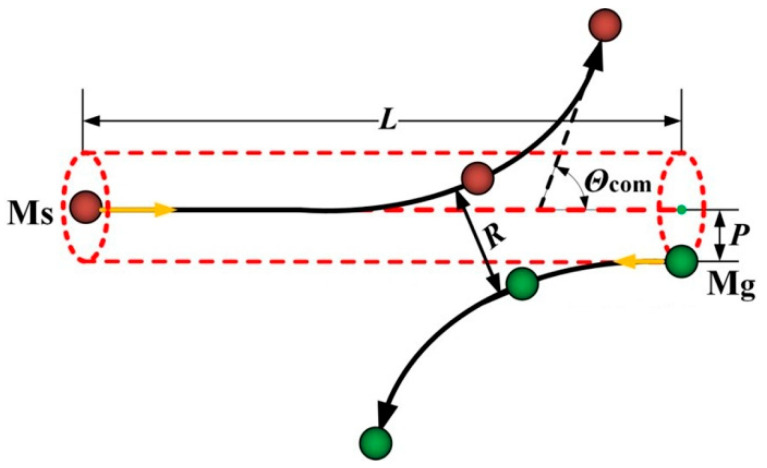
Elastic scattering in the binary collision.

Figure 2 shows a typical binary collision in the center of mass coordinate system. As shown in Figure 1, a sputtered atom (M_s_) collides with a background gas atom (M_g_) with a collision parameter of *p*. The scattering angle of M_s_ after collision is *θ*_com_, which can be determined as [26]:

(10) $$\theta \left(E_{\mathrm{com}},p\right)=\pi -2p\int_{R}^{\infty }1/\left[r^{2}\sqrt{1-U\left(r\right)/E_{\mathrm{com}}-p^{2}/r^{2}}\right]dr$$

(11) $$R=p/\sqrt{1-U_{r}/E_{\mathrm{com}}}$$

(12) $$E_{\mathrm{com}}=m_{s}m_{g}v_{r}^{2}/2\left(m_{s}+m_{g}\right)$$

(13) $$p=P_{\max}\sqrt{R_{3}}$$

where *R* is the closest distance that two colliding atoms can approach; *p* is the actual collision parameter between two colliding atoms; *U*(*r*) is potential function; *R*_3_ is a random number uniformly distributed between 0 and 1. In our simulation, the interaction between the sputtered atom and background gas atom was described by Ziegler–Biersack–Littmark (ZBL) potential [27], which has also been adopted in other MC simulations of sputtered particle transport [28]. Once the *U* (*r*) is determined, for each specific *E*_com_, the function *P*_max_ (*E*_com_) can be evaluated based on Equation (10) by letting *θ*_com_ = 0.01 rad [13]. Then, the actual collision parameter *p* can be calculated by Equation (13), and the actual free path length *λ* can be determined by Equations (6)–(9).

### 2.3. MD Model of the Collision between Sputtered Atom and Background Gas Atom

Since the relative position relationship between M_s_ and M_g_ in the center of mass coordinate system is the same as that in the laboratory coordinate system, the relative position relationship between the two colliding particles shown in Figure 2 can be used to determine the initial positions of the sputtered atom and background gas atom in the laboratory coordinate system during the MD simulation.

**Figure 3:**
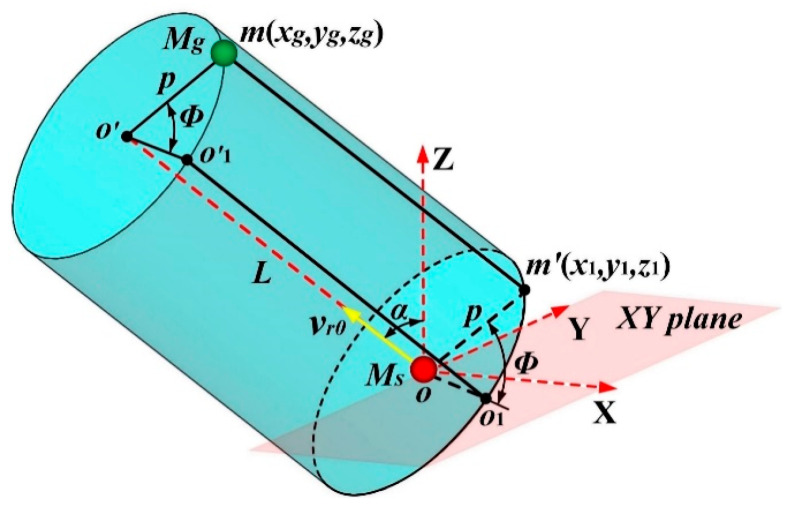
The relative position between the sputtered atom and background gas atom at the initial phase of elastic collision.

Figure 3 shows the initial relative positions between the sputtered atom (M_s_) and background gas atom (M_g_) in three-dimensional space when they collide with a collision parameter of *p*. As shown in Figure 3, the red ball at point *o* (0, 0, 0) represents M_s_, while the green ball at point *m* (*x*_g_*, y*_g_*, z*_g_) denotes M_g_. It can be seen that M_s_ and M_g_ are situated at the bottom surface center and the upper surface edge of an arbitrary spatial cylinder, respectively. The central symmetry axis of the spatial cylinder is along the direction of *v*_r_ (the relative velocity between M_s_ and M_g_), and the radius and height of the spatial cylinder are *p* (collision parameter) and *L* (the distance between M_s_ and M_g_ along the direction of *v*_r_), respectively. In the present simulation, *L* was set to the cutoff radius of the potential function *U*(*r*) in order to save computation time. Then, the initial location of M_g_ can be expressed as:

(14) $$x_{g}=x_{1}+L\cos\alpha_{x}$$

(15) $$y_{g}=y_{1}+L\cos\alpha_{y}$$

(16) $$z_{g}=z_{1}+L\cos\alpha_{z}$$

(17) $$x_{1}^{2}+y_{1}^{2}+z_{1}^{2}=p^{2}$$

(18) $$x_{1}\cos\alpha_{x}+y_{1}\cos\alpha_{y}+z_{1}\cos\alpha_{z}=0$$

(19) $$z_{1}=p\sin\varphi \sin\alpha_{z}\left(0\leq \phi \leq 2\pi \right)$$

where *α*_x_, *α*_y_, and *α*_z_ are the direction angles of *v*_r_ (*v*_rx_, *v*_ry_, *v*_rz_) with respect to the X, Y, and Z coordinate axes, respectively. *Φ* is the azimuth angle of point *m’* (*m*) with respect to the line *oo*_1_ (*o*’*o*’_1_) on the bottom (upper) surface of the spatial cylinder shown in Figure 3, and line *oo*_1_ represents the intersecting line between the XY coordinate plane and the bottom surface of the spatial cylinder. Without losing generality, *Φ =* 2π*R*_4_, where *R*_4_ is a random number distributed uniformly between 0 and 1. Then, the initial position of the background gas atom can be obtained and expressed as:

(20) $$x_{g}=p\left[-v_{\mathrm{rx}}\sin\phi {\cos\alpha }_{z}/\sqrt{v_{\mathrm{rx}}^{2}+v_{\mathrm{ry}}^{2}}+\left|v_{\mathrm{rx}}\right|v_{\mathrm{ry}}\cos\phi /(v_{\mathrm{rx}}\sqrt{v_{\mathrm{rx}}^{2}+v_{\mathrm{ry}}^{2}})\right]+L\cos\alpha_{x}$$

(21) $$y_{g}=p(-v_{\mathrm{ry}}\sin\phi {\cos\alpha }_{z}/\sqrt{v_{\mathrm{rx}}^{2}+v_{\mathrm{ry}}^{2}}-\left|v_{\mathrm{rx}}\right|\cos\phi /\sqrt{v_{\mathrm{rx}}^{2}+v_{\mathrm{ry}}^{2}})+L\cos\alpha_{y}$$

(22) $$z_{g}=p\sin\phi \sin\alpha_{z}+L\cos\alpha_{z}$$

Once the initial locations of two colliding atoms were determined, the velocity (*v*_g_) of the background gas atom can be chosen randomly from the Maxwellian distribution at 300 K, and the initial velocity (*v*_s_) of the sputtered atom can be selected from the Thompson distribution. Then, the elastic collision between the sputtered atom and the background gas atom was simulated by using the molecular dynamics code LAMMPS [29]. The size of the simulation domain was 100 × 100 × 100 Å^3^. Non-periodic and fixed boundary conditions were applied in all three directions in order to capture the entire collision process. The interaction between colliding atoms was described by the Ziegler-Biersack-Littmark (ZBL) potential [27] with a cut-off distance of 6.2 Å [30]. At the beginning of the simulation, the sputtered atom was placed at the geometric center of the simulation box (coordinate origin), while the background gas atom was put on the coordinates calculated according to Equations (20)–(22). The standard Velocity-Verlet algorithm was used for integrating Newton’s equation of motion. The time step was set to 0.1 fs [18]. The MD simulation was terminated when the force exerted on the sputtered atom went to zero. Then, the velocity data of the sputtered atom was written into a dump file, which would be read during the MC simulation.

### 2.4. MC-MD Coupled Simulation Scheme

**Figure 4:**
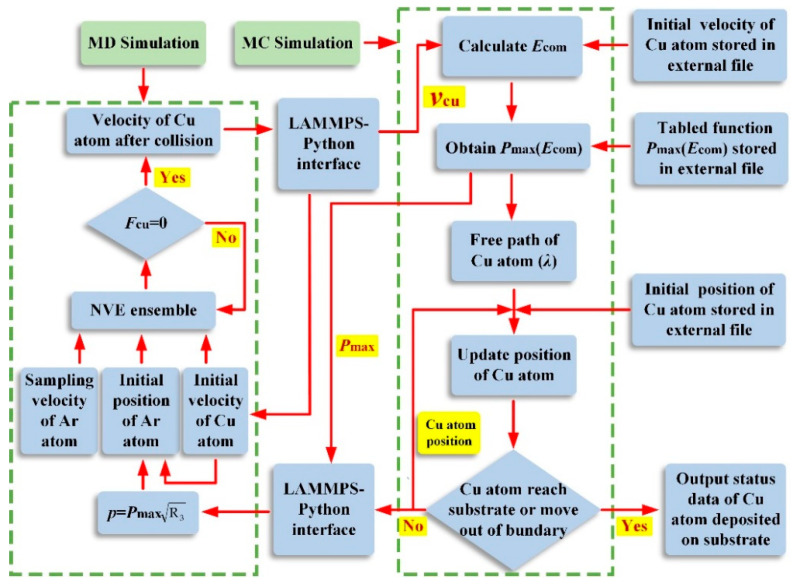
The flow charts of the MC-MD coupled simulation.

In this study, the MC-MD coupling simulation of sputtered particle transport was realized by the LAMMPS input script program, where the LAMMPS-Python interface was used to invoke the python functions for the MC simulation defined in external files. Specifically, the MD simulation of the elastic collision between the sputtered atom and the background gas atom was performed to calculate the post-collision velocity of the sputtered atom, which was written into a dump file. The MC simulation of the free-flight process of the sputtered atom was conducted by Python functions, which were defined to calculate the free path of the sputtered atom and update the location of the sputtered atom according to the velocity data read from the dump file. The data exchange between the MD and MC simulations was realized via the LAMMPS-Python interface. In this MC-MD coupling simulation, one sputtered Cu atom was tracked at each simulation loop in the three-dimensional space between the target and substrate until it arrived at the substrate or escaped from the monitoring space, and then the next sputtered Cu atom was emitted and followed. This procedure was repeated until a total of 1,000,000 Cu atoms were released. The flow charts of the MC-MD coupling simulation are shown in Figure 4.

## 3. Reliability Verification of the MD Collision Model

**Figure 5:**
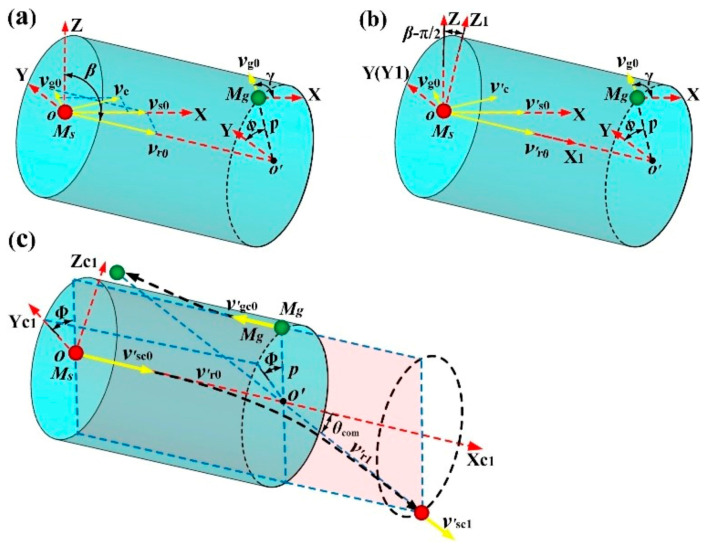
Scattering collision between sputtered Cu atom (M_s_) and background gas atom (M_g_) with specific initial velocities. (**a**) Initial velocities and positions of M_s_ and M_g_ in the laboratory coordinate system XYZ; (**b**) initial velocities and positions of M_s_ and M_g_ in the laboratory coordinate system X_1_Y_1_Z_1_; (**c**) scattering collision between M_s_ and M_g_ in the center of mass coordinate system X_c1_Y_c1_Z_c1_ when the coordinate system X_1_Y_1_Z_1_ floats with the mass center of colliding atoms.

In order to validate the reliability of the MD collision model proposed in our work, an analytical model was established to calculate the scattering angle of the sputtered Cu atom after colliding with the Ar atom. In this analytical model, the initial velocities of Cu and Ar atoms before collision were both within the XZ plane of the laboratory coordinate system, and the initial energy of Cu and Ar atoms were set to 1 eV and 0.0387 eV (the average energy of Ar atom at 300 K), respectively. Figure 5 displays the scattering process of the Cu atom in this specific collision. In Figure 5a, the initial velocities of the Cu atom and Ar atom are *v*_s0_ (*v*_sx_, 0, 0) and v_g0_ (*v*_gx_, 0, *v*_gz_), respectively; the angle of initial relative velocity *v*_r0_ with respect to the Z coordinate axis is *β*; the angle of *v*_g0_ with respect to the X coordinate axis is *γ*; *v*_c_ is the center-of-mass velocity.

To facilitate analysis, another laboratory coordinate system X_1_Y_1_Z_1_ was established, as shown in Figure 5b. In the coordinate system X_1_Y_1_Z_1_, the X_1_ and Y_1_ coordinate axes are along the directions of *v*_r0_ and the Y axis of coordinate system XYZ, respectively. Consequently, the initial velocity of the Cu atom and the center-of-mass velocity in the coordinate system X_1_Y_1_Z_1_ can be expressed by the following equations:

(23) $${v^{\prime }}_{s0}=\left[\begin{array}{c} v_{\mathrm{sx}}\sin\beta \\ 0\\ -v_{\mathrm{sx}}\cos\beta \end{array}\right]$$

(24) $${v^{\prime }}_{c}=\left[\begin{array}{c} v_{\mathrm{cx}}\sin\beta +v_{\mathrm{cz}}\cos\beta \\ 0\\ -v_{\mathrm{cx}}\cos\beta +v_{\mathrm{cz}}\sin\beta \end{array}\right]$$

In the center of mass coordinate system X_c1_Y_c1_Z_c1_, i.e., the coordinate system X_1_Y_1_Z_1_ floating with the mass center of colliding atoms, the scattering process of the Cu atom after colliding with the Ar atom can be displayed in Figure 5c. Since angular momentum and energy are conserved and potential is just determined by the distance between the colliding atoms, the motion of the colliding atoms in the center of mass coordinate system has the following characteristics: (1) the relative velocity between Cu and Ar atoms in the center of mass coordinate system is identical to that in the laboratory coordinate system; (2) the movement of Cu atom is restricted in a plane [26]; (3) the velocity magnitude of Cu atom remains constant before and after collision. Accordingly, as shown in Figure 5c, the post-collision velocity of the Cu atom in the center of mass coordinate system X_c1_Y_c1_Z_c1_ can be expressed as follows:

(25) $${v^{\prime }}_{sc1}=\frac{m_{g}\left|v_{r0}\right|}{m_{g}+m_{s}}\left[\begin{array}{c} \cos\theta_{\mathrm{com}}\\ -\sin\theta_{\mathrm{com}}\cos\Phi \\ -\sin\theta_{\mathrm{com}}\sin\Phi \end{array}\right]$$

where |*v*_r0_| is the magnitude of *v*_r0_; *Φ* is the initial position azimuth angle of the Ar atom in the Y_c1_Z_c1_ plane. Therefore, in the laboratory coordinate system X_1_Y_1_Z_1_, the post-collision velocity of the Cu atom can be expressed by:

(26) $${v^{\prime }}_{s1}=\left[\begin{array}{c} v_{\mathrm{cx}}\sin\beta +v_{\mathrm{cz}}\cos\beta +m_{g}\left|v_{r0}\right|\cos\theta_{\mathrm{com}}/\left(m_{s}+m_{g}\right)\\ -m_{g}\left|v_{r0}\right|\sin\theta_{\mathrm{com}}\cos\Phi /\left(m_{s}+m_{g}\right)\\ -v_{\mathrm{cx}}\cos\beta +v_{\mathrm{cz}}\sin\beta -m_{g}\left|v_{r0}\right|\sin\theta_{\mathrm{com}}\sin\Phi /\left(m_{s}+m_{g}\right) \end{array}\right]$$

The scattering angle of the Cu atom in the laboratory coordinate system can be expressed as:

(27) $$\theta_{lab}=\arccos\frac{v_{\mathrm{cx}}+{\left(2\eta eE_{\mathrm{com}}/m_{s}\right)}^{1/2}(\cos\theta_{\mathrm{com}}\sin\beta +\sin\theta_{\mathrm{com}}\cos\beta \sin\Phi )}{\sqrt{\begin{array}{l} 2\eta eE_{\mathrm{com}}/m_{s}+{\left|v_{c}\right|}^{2}+2({2\eta eE_{\mathrm{com}}/m_{s})}^{1/2}\sin\theta_{\mathrm{com}}(v_{\mathrm{cx}}\sin\beta +v_{\mathrm{cz}}\cos\beta )\\ -2({2\eta eE_{\mathrm{com}}/m_{s})}^{1/2}\sin\theta_{\mathrm{com}}(v_{\mathrm{cz}}\sin\beta -v_{\mathrm{cx}}\cos\beta )\sin\Phi \end{array}}}$$

where *η* = *m*_g_/(*m*_g_ + *m*_s_); *e* denotes the charge of electron. Indeed, *β* is only determined by the initial velocity direction of the Ar atom (*γ*) as the initial velocity of the Cu atom is along the X axis, and *θ*_com_ is only determined by the collision parameter *p* as *E*_com_ is kept constant. Therefore, from Equation (27), it can be seen that *θ*_lab_ is influenced by the initial velocity direction of the Ar atom (*γ*), collision parameter *p,* and the initial position azimuth angle of the Ar atom (*Φ*).

As discussed in Section 1, to avoid complex coordinate transformation, an approximate formula is normally used to calculate the scattering angle of the sputtered atom after collision, which can be expressed as:

(28) $$\tan\theta_{\mathrm{lab}}=\sin\theta_{\mathrm{com}}/\left(\cos\theta_{\mathrm{com}}+m_{s}/m_{g}\right)$$

Since Equation (28) is derived by supposing that the background gas atom is stationary before collision, the effect of *γ* and *Φ* on the scattering angle of the sputtered atom after collision cannot be considered in Equation (28).

The reliability of the MD collision model and the computation error of Equation (28) will be discussed in detail below.

### 3.1. Influence of the Initial Velocity Direction of Ar Atom on θ_lab_

**Figure 6:**
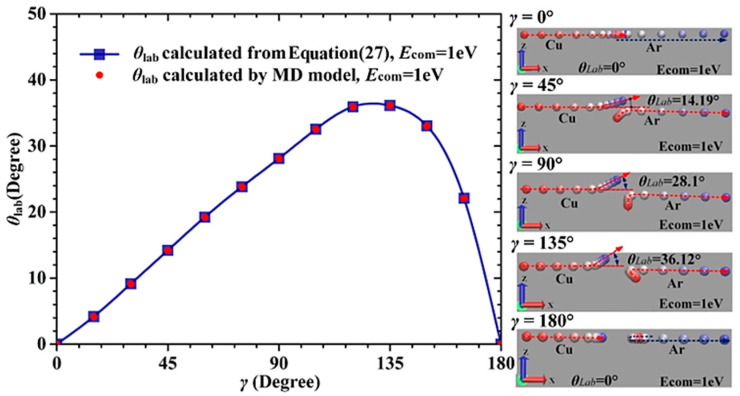
Influence of the initial velocity direction of the Ar atom on the scattering angle of the Cu atom (*E*_com_ = 1 eV).

Figure 6 shows the variation of *θ*_lab_ with the initial velocity direction of the Ar atom when *E*_com_, *p* and *E*_Ar_ are kept at 1 eV, 0 Å, and 0.0387 eV, respectively. As shown in Figure 6, the values of *θ*_lab_ calculated by the MD collision model are consistent well with those calculated by Equation (27), which validates the reliability of the MD collision model. When *E*_com_ = 1 eV and *p* = 0 Å, *θ*_lab_ increases first and then decreases as *γ* increases from 0° to 180°, and the maximum value of *θ*_lab_ is 36.12°. Accordingly, the initial velocity direction of the background gas atom significantly affects the scattering angle of the sputtered atom, but this effect cannot be considered in Equation (28).

### 3.2. Influence of Collision Parameter p on θ_lab_

**Figure 7:**
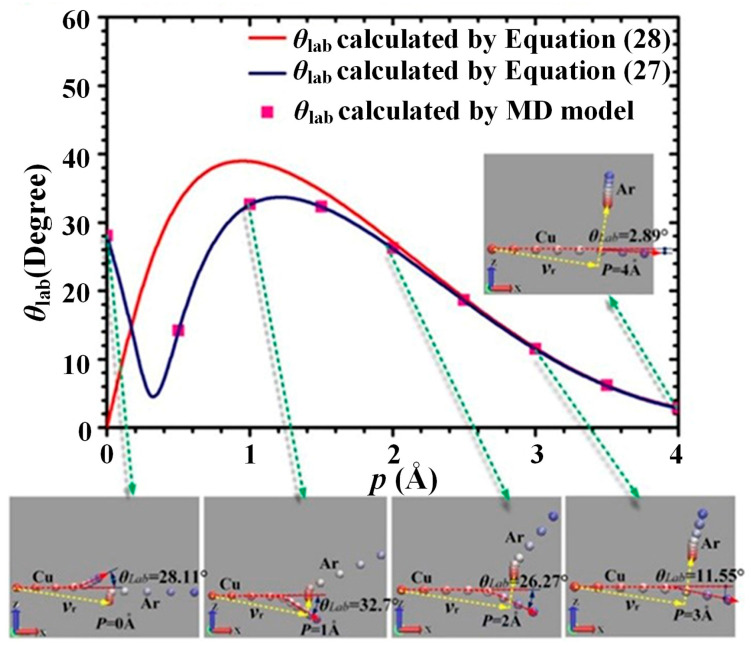
Influence of the collision parameter *p* on the scattering angle of Cu atom when *Φ* = 90° (*E*_com_ = 1 eV).

Figure 7 shows the influence of collision parameter *p* on *θ*_lab_ when *Φ, E*_com_, *E*_Ar_, and *γ* are kept at 90°, 1 eV, 0.0387 eV, and 90°, respectively. In Figure 7, the values of *θ*_lab_ were calculated individually by Equation (27), Equation (28), and the MD collision model as *p* increased from 0 to 4 Å. As shown in Figure 7, as *p* increases from 0 to 4 Å, the values of *θ*_lab_ calculated by the MD collision model agree well with the corresponding values of *θ*_lab_ calculated by Equation (27), while significant errors exist in the values of *θ*_lab_ (*p* < 2 Å, *E*_com_ = 1 eV) calculated by Equation (28).

### 3.3. Influence of the Initial Position Azimuth Angle of Ar Atom on θ_lab_

**Figure 8:**
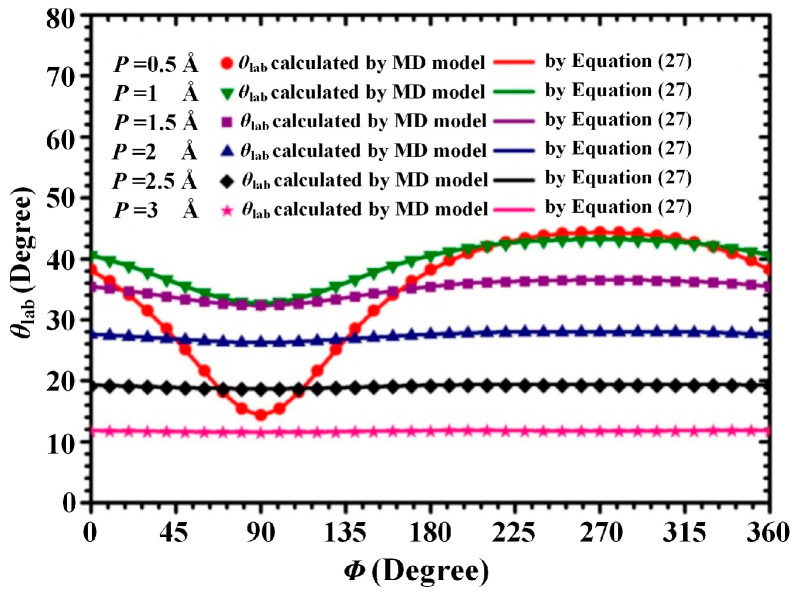
Influence of the initial position azimuth angle of an Ar atom on θ_lab._ (*E*_com_ = 1 eV).

Figure 8 shows *θ*_lab_ as a function of the initial position azimuth angle of the Ar atom (*Φ*) when *E*_com_, *E*_Ar_ and *γ* are kept at 1 eV, 0.0387 eV, and 90°, respectively. In Figure 8, the values of *θ*_lab_ were calculated individually by Equation (27) and MD collision model as *Φ* ranged from 0° to 360° and *p* increased from 0 to 3 Å. As shown in Figure 8, the values of *θ*_lab_ calculated by the MD collision model are coincident with the corresponding values of *θ*_lab_ calculated by Equation (27). As shown in Figure 8, when *E*_com_= 1 eV and 0.5 Å ≤ *p* ≤ 1.5 Å, the values of *θ*_lab_ change significantly as *Φ* increases from 0° to 360°. Accordingly, the initial position azimuth angle of the background gas atom (*Φ*) exerts a significant influence on the scattering angle of the sputtered atom. However, this influence cannot be considered in Equation (28).

## 4. Results and Discussion

### 4.1. Example of the Transport Process of a Sputtered Atom in Gas Phase

**Figure 9:**
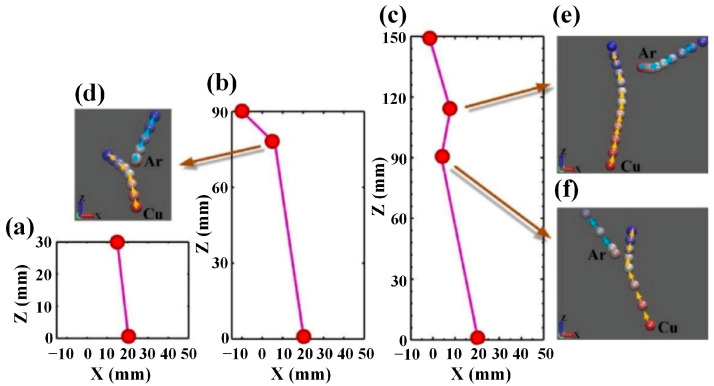
Trajectories of a sputtered atom in the gas phase at the target-substrate distances of (**a**) 30 mm, (**b**) 90 mm, and (**c**) 150 mm and the dynamic evolution of the elastic collisions between Cu and Ar atoms at the target-substrate distances of (**d**) 90 mm and (**e**,**f**) 150 mm. In sub-graphs (**a**–**c**), red balls denote the locations of the Cu atom, and purple lines represent the free flight trajectories of the Cu atom. In sub-graphs (**d**–**f**), the positions of Cu and Ar atoms at different time steps are presented by two strings of colored balls marked by yellow and blue arrows, respectively.

Figure 9 displays the transport processes of an identical sputtered Cu atom in argon gas when the target-substrate distance is set to 30, 90, and 150 mm, respectively. Figure 9a–c depicts the macroscopic trajectories of the sputtered Cu atom, while Figure 9d–f show the microscopic evolution of elastic collisions between Cu and Ar atoms. As shown in Figure 9a, at the target-substrate distance of 30 mm, the sputtered Cu atom is directly deposited onto the substrate surface without being subjected to any collision. As shown in Figure 9b,c, at the target-substrate distances of 90 and 150 mm, the sputtered Cu atom undergoes one or more scattering collisions before it ultimately arrives at the substrate surface since the target-substrate distance exceeds the free path of the sputtered Cu atom. From the microscopic trajectory of the sputtered atom shown in Figure 9d–f, it can be seen that the spacing between the two adjacent positions gradually reduces as time goes on, while the case for the trajectory of the Ar atom is just reversed. This means that the kinetic energy of the sputtered Cu atom decreases while that of the Ar atom increases, which indicates the occurrence of energy transfer between Cu and Ar atoms. Furthermore, comparing Figure 9d with Figure 9b and Figure 9e,f with Figure 9c, respectively, it can be found that the variations in the motion direction of the Cu atom on the microscopic scale are consistent with those in the macroscopic scale, which suggests that the coupling of the MD and MC simulations has been successfully implemented.

### 4.2. Incident Energy Distribution

**Figure 10:**
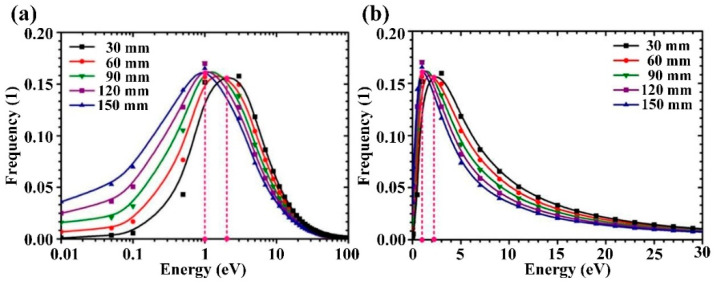
Incident energy distribution of the deposited sputtered Cu atoms at different target-substrate distances plotted on (**a**) logarithmic scale and (**b**) linear scale.

The incident energy of sputtered Cu atom striking the substrate surface is a key parameter that influences the crystalline microstructure [31] and grain size [32] of the sputtered film. Figure 10 displays the incident energy distributions of sputtered Cu atoms under different target-substrate distances. To clearly display the incident energy distributions in the low energy regime (*E* < 1 eV) and moderate energy regime (1 eV < *E* < 30 eV), the distribution curves were plotted on both logarithmic and linear scales. As target-substrate distance increases from 30 to 150 mm, the proportion of the deposited atoms with incident energy less than 1 eV gradually increases, while the fraction of the deposited atoms with incident energy ranging from 2 to 30 eV significantly decreases, and the peak energy of kinetic energy distribution gradually shifts from 2 to 1 eV. This indicates that, as target-substrate distance increases, more sputtered atoms with moderate kinetic energy (i.e., of the order of 10 eV) are gradually thermalized during the transport process in the gas phase, which is due to the fact that the increased target-substrate distance is gradually equal to or greater than the free paths of these sputtered atoms. As shown in Figure 10b, the incident energy distribution of deposited Cu atoms resembles a Maxwellian-type distribution with a peak energy of about 1–2 eV and a high-energy tail extending to 30 eV, which is consistent with the experimental results and theoretical calculation results reported in Ref. [25] and Ref. [33], respectively.

**Figure 11:**
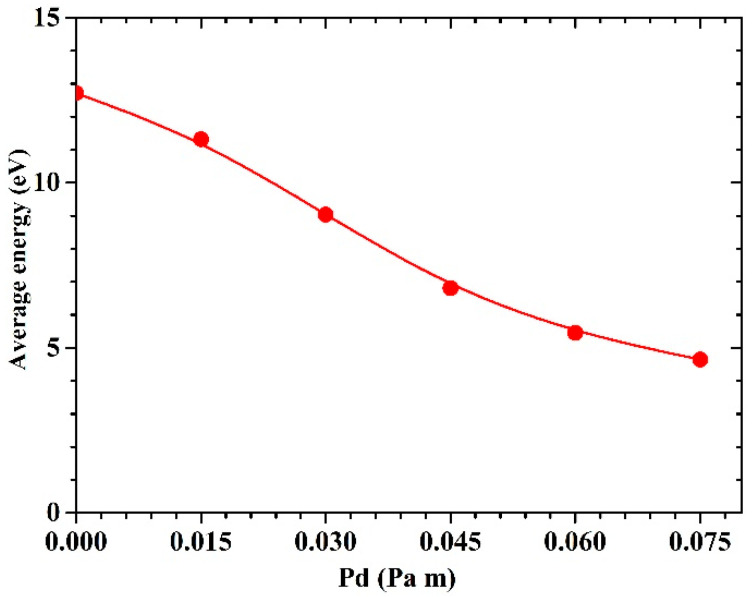
The average energy of sputtered atoms as a function of Pd values.

The average energy of deposited Cu atoms is an important parameter to characterize the energy deposition onto the substrate. Figure 11 shows the values of average energy calculated by the MC-MD simulation under different Pd values (the product of pressure and target substrate distance). It can be seen that, as the Pd value increases from 0 Pa m to 0.075 Pa m, the values of average energy calculated undergo an approximately exponential attenuation. In addition, the reduction of average energy is more significant as the Pd value increases from 0.015 Pa m to 0.045 Pa m. This might be mainly due to the fact that some sputtered atoms in the energy range of 2 to 30 eV (approximately account for 70% of total sputtered Cu atoms) are subjected to at least one elastic collision and gradually lose their energy since their mean free paths range from 41.6 to 113.7 mm and their energy loss in the first few collisions is normally greater than that in the subsequent collisions. The average energy values of Cu atoms arriving at the substrate are higher than those calculated in Ref. [10] and Ref. [34]. In our MC-MD simulation, energy-dependent collision cross-sections were used. Therefore, the mean free path of sputtered atoms is significantly greater at higher energies than that used in Ref. [34], which results in less energy loss of Cu atoms. Furthermore, the maximum energy in the initial energy distribution of Cu atoms in Ref. [10] and Ref. [34] was set to 40 eV. This artificial limitation for the upper-limit energy in the initial energy distribution improves computational efficiency but leads to the lower average energy of sputtered atoms [33].

### 4.3. Incident Angle Distribution

The incident polar angle distribution of the deposited atom significantly influences the surface topography of the sputtered film [35]. Herein, the incident polar angle denotes the angle with respect to the normal substrate surface. To calculate the incident polar angle distribution of deposited atoms, the range of polar angle was partitioned into 19 divisions, including two divisions of 2.5° (0–2.5° and 87.5–90°) and 17 divisions of 5° covering the range of 2.5° to 87.5°. Then, the incident polar angle distributions were obtained by calculating the proportions of the deposited atoms clarified into the 19 polar angle divisions, regardless of the incident azimuth angle of the deposited atoms. Therefore, the incident polar angular distribution can be considered as the distribution of deposition yield within the 19 element solid angles.

**Figure 12:**
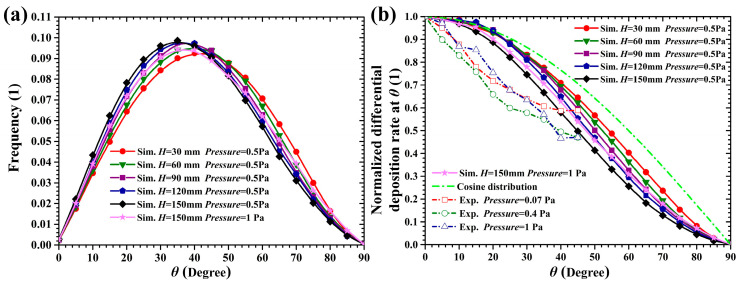
Incident angle distribution of sputtered Cu atoms under different target-substrate distances, (**a**) incident polar angle distribution; (**b**) normalized differential deposited rate as a function of polar angle obtained by simulation and experiment [38].

Figure 12a shows the incident polar angle distributions of sputtered atoms under different target-substrate distances, which are typical arch-shaped profiles [11,12,15,16]. As the PD value (the product of gas pressure and target-substrate distance) increases from 0.015 to 0.075 Pa m, the peak angle of the incident polar angle distribution decreases from 45° to 35° and then shifts toward to 45° at the PD value of 0.15 Pa m (*H* = 150 mm, *Pressure* = 1 Pa). This increasingly forward-peaked distribution of incident polar angle might be attributed to the scattering collision experienced by the sputtered Cu atoms whose free paths are approximately equal to or less than the target-substrate distance. Indeed, the sputtered atoms, which were ejected with a small polar angle with respect to the normal target surface, are prone to arrive at the substrate surface without undergoing any scattering collision due to the comparatively small line-of-sight distance from their emission position to the substrate surface. On the contrary, the sputtered atoms emitted with a large polar angle would have a higher possibility of undergoing scattering collision and either arrive at the substrate surface with a smaller incident angle or escape from the space between the target and substrate with a larger flying polar angle. Therefore, with the increase of target-substrate distance, the amount of the Cu atoms striking the substrate with small incident polar angles (<45°) increases. This indicates the selectivity of scattering collision to the incident polar angle of the Cu atoms deposited onto the 2-inch substrate surface. On the other hand, at the PD value of 0.15 Pa m, the mean collision number of the sputtered atom with the energy of 100 eV (90% of sputtered atoms possess energy less than 100 eV) is 1.58, which indicates that almost all the sputtered atoms undergo elastic scattering in the gas phase. Due to this significant gas scattering effect, the incident angle distribution would gradually approach the cos*θ*sin*θ* distribution with a peak incident angle of 45°, i.e., the sputtered atoms are gradually thermalized and have an isotropic velocity distribution [36,37].

The angle distribution of sputtered Cu atoms was also experimentally measured [38] by sampling the differential deposition rate (The yield per unit solid angle, i.e., *N*i/△Ω) at an interval of 5° in the polar angle range of 0° to 45°. In order to compare the experimental measured solid angle distribution with our simulation results, the incident polar angle distributions shown in Figure 12a should be transformed based on Equation (5):

(29) $$\Delta N_{i}=N_{t}f_{E}(\theta )\Delta \Omega =N_{t}f_{s}(\theta )\Delta \theta$$

(30) $$f_{E}(\theta )=f_{s}(\theta )\Delta \theta /\Delta \Omega =f_{s}(\theta )/\left(2\pi \sin\theta \right)$$

where Δ*N*i is the number of sputtered atoms in the solid angle of 5° (△*Ω*); *θ* is the polar angle corresponding to △*Ω*; *f*_s_(*θ*) is the incident angle distribution of sputtered atoms as shown in Figure 12a; *f*_E_(*θ*) represents the distribution of differential deposition rate in unit solid angle. Herein, *θ* was set to 0.5° in the calculating *f_E_*(*θ = *0°) such that sin*θ* is not equal to 0 in Equation (30).

Figure 12b shows the normalized distribution of differential deposition rate in unit solid angle displayed in Figure 3 of [38] and those transformed from the incident polar angle distributions shown in Figure 12a. In Figure 12b, all the probability density values are normalized by those at the polar angle of 0°, i.e., normal to the target surface. Due to the difference in process conditions (PD values) between our simulation and the experiment in Ref. [38], the distribution curves obtained by simulation calculation cannot be exactly consistent with those measured by the experiment, but the distribution curves derived by the two approaches are similar in shape. In addition, as the PD value increases from 0.015 to 0.075 Pa m, the distribution curve of differential deposition rate in unit solid angle becomes more forward-peaked, while as the PD value further increases to 0.15 Pa m (*Pressure* = 1 Pa, *H* = 150 mm), the distribution curve changes reversely, approaching the cosine distribution. These changes in the calculated distribution curve with the increase of PD value agree with the variation of experimentally measured distribution in Figure 3 of Ref. [38]. It suggests that our simulation results can correctly reveal the effect of the PD value on the incident angle distribution of deposited atoms.

## 5. Conclusions

An MC-MD coupling simulation scheme for sputtered particle transport in DC magnetron sputtering discharge was first proposed in this work. In this MC-MD simulation method, the MD simulation was performed by using LAMMPS to simulate the elastic collision between the sputtered atom and background gas atom, while the MC simulation was conducted by using Python to model the free-flight process of sputtered atoms. The MC-MD coupling simulation method was employed to investigate the incident energy and polar angle distributions of deposited Cu atoms under different target-substrate distances. As the target-substrate distance increased from 30 to 150 mm, the peak energy of incident energy distribution decreased from 2 to 1 eV, which might be due to the thermalization of the sputtered atoms with moderate energy (i.e., of the order of 10 eV). The average energy of deposited Cu atoms under different PD values underwent an approximately exponential attenuation. As the PD value increased from 0.015 to 0.075 Pa m, the peak angle of the incident polar angle distribution decreased from 45° to 35°, which might result from the selectivity of scattering collision to the incident angle of deposited Cu atoms. As the PD value further increased to 0.15 Pa m, the incident polar angle distribution of sputtered atoms changed reversely and approached the cos*θ*sin*θ* distribution, which might be caused by the gradual thermalization of sputtered atoms with the increase of target-substrate distance. This work provides a scale-matched simulation method for sputtered particle transport. More important, the MC-MD coupling simulation script, which is executed on the soft platform of LAMMPS, can be conveniently expanded as a combined simulation program for the sputtered atoms transport and deposition processes so as to explore the influence mechanism of process parameters on the properties of sputtered film.

## Data Availability

Not applicable.
